# A232 TUFT CELLS COORDINATE RAPID EXPULSION OF THE TAPEWORM *H. DIMINUTA* BUT ARE NOT REQUIRED FOR ENHANCED IMMUNITY AGAINST THE NEMATODE, *H. POLYGYRUS,* IN MICE PREVIOUSLY INFECTED WITH *H. DIMINUTA*

**DOI:** 10.1093/jcag/gwab049.231

**Published:** 2022-02-21

**Authors:** S Rajeev, A Leon-Coria, A Wang, C Finney, D M Mckay

**Affiliations:** 1 University of Calgary Department of Biological Sciences, Calgary, AB, Canada; 2 University of Calgary Cumming School of Medicine, Calgary, AB, Canada

## Abstract

**Background:**

The tuft cell is an important sentinel that monitors the gut lumen and coordinates immunity against parasitic nematodes. We showed small intestinal tuft cell hyperplasia 11 days post-infection (dpi.) with the tapeworm *Hymenolepis diminuta*: a time when the parasite is no longer present in murine hosts. This may be a way by which the host protects itself from subsequent helminth-infections, a common phenomenon in parasite-endemic world regions. We test this supposition using Pou2f3^-/-^ mice that lack tuft cells.

**Aims:**

To test the hypothesis that tuft cells are important in the anti-worm response in *H. diminuta* ( *H.d.*)-infected mice subsequently infected with the nematode parasite *Heligosomoides polygyrus* ( *H.p.*).

**Methods:**

Male C57BL6 and Pou2f3^-/-^ mice (8–12 weeks) were infected with 5 *H.d.* cysticercoids ± 200 *H.p.* larvae at 10 dpi with *H. diminuta* (non *H.p.* mice - control). Upon necropsy at 24 dpi *H. diminuta* (i.e. 14 dpi *H.p.* in co-infected mice), both worms were ennumerated in small intestinal washings, *H.p.* granulomas examined and fecal egg counts performed. Small intestinal segments were stained for tuft (DCLK1^+^) and goblet cells (PAS^+^). As a surrogate of successful infection, IL-4 and IL-10 were measured in supernatants from concanavalin-A treated splenocytes.

**Results:**

Wild-type (WT) mice expel *H. diminuta* by 11 dpi and this was delayed in Pou2f3^-/-^mice, with worms readily detectable at 14 dpi and absent by 21 dpi. Despite the delayed expulsion, both WT and Pou2f3^-/-^ mice showed increased splenic production of IL-4 and IL-10; however, unlike WT mice, *H. diminuta*-infected Pou2f3^-/-^ mice show no increase in jejunal goblet cell numbers. Mice infected with *H. diminuta* displayed a degree of increased resistance to *H.p*.-infection defined by reduced worm and egg burdens, and increased granuloma formation in comparison to *H.p.*-only infected animals. In this sequential co-infection model, there were no significant differences between WT and Pou2f3^-/-^ mice in the response to *H.p*.

**Conclusions:**

The absence of tuft cells slows expulsion of *H. diminuta* from its non-permissive mouse host and correlates with diminished goblet cell hyperplasia. Hypothesizing that *H. diminuta*-evoked tuft cell hyperplasia would enhance the immune response to a subsequent infection with an unrelated nematode parasite proved incorrect. While *H. diminuta*-infected mice were partially protected from *H.p*., response was similar in WT and Pou2f3^-/-^ mice. Thus tuft cells are important in worm detection: yet, our co-infection data suggests that other events initiated by the primary worm infection impact the outcome of subsequent infection with a different helminth and tuft cells have a limited, if any, role to play in this helminth-host-helminth interaction.

**Funding Agencies:**

CIHRNSERC, Eye’s High International doctoral scholarship

